# Comparison of Different Electricity-Based Thermal Pretreatment Methods for Enhanced Bioenergy Production from Municipal Sludge

**DOI:** 10.3390/molecules23082006

**Published:** 2018-08-11

**Authors:** E. Hosseini Koupaie, T. Johnson, C. Eskicioglu

**Affiliations:** UBC Bioreactor Technology Group, School of Engineering, The University of British Columbia Okanagan Campus, 1137 Alumni Avenue, Kelowna, BC V1V 1V7, Canada; ehssan.hosseini.k@gmail.com (E.H.K.); thomas.johnson@ubc.ca (T.J.)

**Keywords:** anaerobic digestion, bioenergy, municipal sludge, solubilization, thermal pretreatment

## Abstract

This paper presents results for a comprehensive study that compares the performance of three electricity-based thermal pretreatment methods for improving the effectiveness of anaerobic digestion (AD) to process municipal wastewater sludge. The study compares thermal pretreatment using conventional heating (CH), microwave (MW), and radio frequency (RF) heating techniques. The effectiveness of the pretreatment methods was assessed in terms of chemical oxygen demand (COD) and biopolymers solubilization, AD bioenergy production, input electrical energy, and overall net energy production of the sequential pretreatment/AD process. The heating applicators for the bench-scale testing consisted of a custom-built pressure-sealed heating vessel for CH experiments, an off-the-shelf programmable MW oven operating at a frequency of 2.45 GHz for MW heating experiments, and a newly developed 1 kW RF heating system operating at a frequency of 13.56 MHz for RF heating experiments. Under identical thermal profiles, all three thermal pretreatment methods achieved similar sludge disintegration in terms of COD and biopolymer solubilization as well as AD bioenergy production (*p*-value > 0.05). According to the energy assessment results, the application of CH and MW pretreatments resulted in overall negative energy production, while positive net energy production was obtained through the sequential pretreatment/AD process utilizing RF pretreatment.

## 1. Introduction

Municipalities rely on physical, chemical, and biological treatment processes to treat their municipal and industrial wastewater. As a result of these treatment processes, municipal sludge, a by-product of treatment, is generated in wastewater treatment plants (WWTP). Currently, about 0.7 million tons of dry municipal sludge are produced annually in Canada [1]. In the United States and Europe sludge volumes are even higher, and annual production ranges from 7 to 10 million [2,3]. To service the demands of growing cities and respond to the increasingly stringent wastewater regulations, existing treatment plants are expanding, resulting in increased production of municipal sludge. Therefore, the management of wastewater residual sludge has now become one of the world’s largest and most critical management challenges.

Among different sludge handling/disposal methods (i.e., incineration, composting, and landfilling), AD has aroused more attention in recent years due to its potential for generating renewable energy in the form of methane gas. In addition to the bioenergy production, the cost of the moving, handling, and processing the waste sludge is minimized due to the significant volume reduction after the AD process [4]. AD is a complex biochemical process comprised of four main sequential stages: hydrolysis, acidogenesis, acetogenesis, and methanogenesis [5]. Of the four stages, the hydrolysis stage is known as a rate-limiting stage because high molecular-weight organics are converted into low molecular-weight or soluble compounds [6]. In terms of the municipal sludge, the hydrolysis stage is particularly limited due to a high content of microbial cells and extracellular polymeric substances resisting enzymatic reactions [7]. 

Previous research has proven that thermal pretreatment (hydrolysis) can accelerate the digestion process by increasing the soluble fraction of organic matter before AD. The thermal pretreatment methods primarily use conventional (conductive) heating (CH) or microwave (MW) irradiation [8,9,10,11]. In CH, the heat transfer mechanism is through a thermal conduction process where energy is transferred from more energetic to less energetic particles due to the thermal gradient [12]. The thermal gradient can lead to non-uniform heating as well as a transient thermal lag throughout the load. As a way of overcoming the limitations of CH, during the last decade, more attention has been given to the application of MW heating at a frequency of 2.45 GHz for sludge hydrolysis [9,11,13,14,15]. Unlike CH, in MW heating, the electric field interacts directly with molecules in the load and increases kinetic energy to heat the load. The main drawbacks of MW heating is the short penetration depth of the electromagnetic waves which creates non-uniform heating throughout the load and the low energy efficiency (~60%) of high power MW generators [16].

Studies have been conducted to compare the effects of CH and MW pretreatment methods for enhanced sludge solubilization and AD performance. From these studies, some researchers concluded that MW heating is a more effective than CH because of athermal (non-thermal) effects where the electric field intensity directly damages cellular structures rather than through thermal effects [14,15,17,18,19,20,21]. However, other researchers have concluded that CH is a better thermal pretreatment method compared to MW heating [9,11,13,14]. In other studies, no significant differences between CH and MW pretreatments have been measured with respect to sludge solubilization or biogas production [9,17,22,23]. From these studies, it is difficult to draw a conclusion on the merits of CH and MW heating due to the contradictory observations. Additionally, most of these studies were limited to the performance evaluation of the CH and MW systems without conducting an energy assessment and this motivates further study.

In this paper, a third thermal pretreatment method using RF heating was added to a comparative study of CH and MW heating. The RF heating system was a custom heating apparatus that was specifically designed to efficiently heat municipal sludge based on the electrical properties of the load. Experiments were conducted to compare the three electricity-based thermal pretreatment methods (CH, MW, and RF). Measurements were made to quantify the disintegration of municipal sludge and determine the bioenergy production from the AD process. The electrical energy required for each thermal pretreatment process was also measured to calculate the overall energy efficiency of the thermal pretreatment process, and conclusions on energy efficiency for the three methods are summarized.

## 2. Results and Discussion

### 2.1. Comparison of CH, MW, and RF Pretreatments for Sludge Disintegration

**Figure 1:**
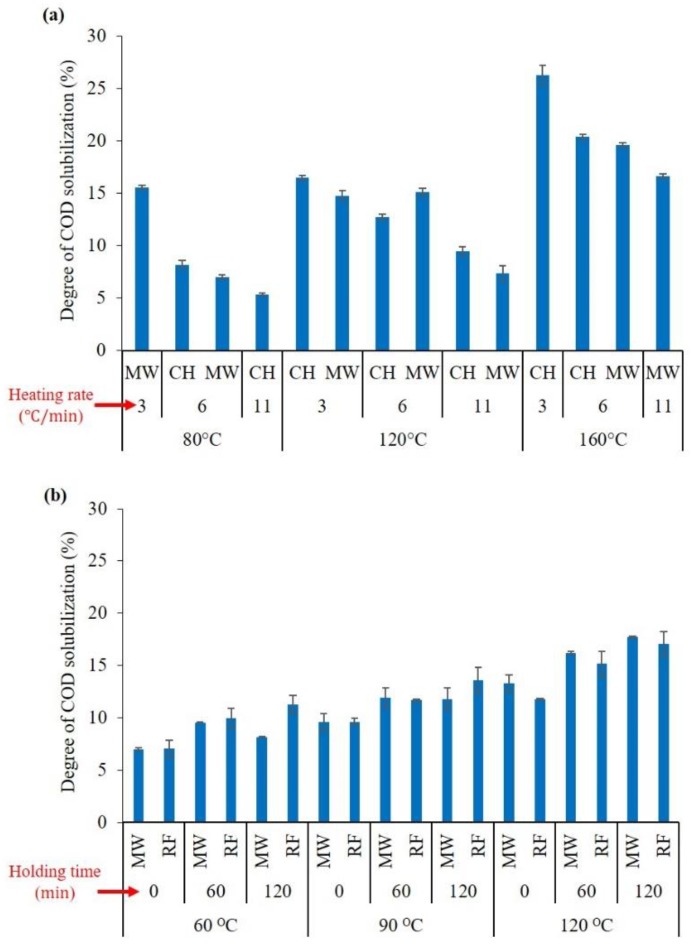
Comparison of CH, MW, and RF pretreatments for the solubilization of COD from; (**a**) CH vs. MW study and (**b**) MW vs. RF study.

Figure 1a compares the degree of COD solubilization for the CH and MW pretreatment systems under different temperatures and heating rates. According to Equation (1), the degree of solubilization (DS) represents the percentage of the substrate (in terms of COD, sugar, protein, and humic acid) that is converted from the particulate to soluble phase during the pretreatment.

(1) $$\;\mathrm{DS}\;\left(\%\right)=100\times \frac{\left(S_{2}-S_{1}\right)}{\left(T_{1}-S_{1}\right)}$$

where, $S_{1}$ and $T_{1}$ are the concentration of the soluble and total fraction before pretreatment, respectively and $S_{2}$ is the concentration of the soluble fraction after pretreatment (in mg/L).

As expected, regardless of the thermal pretreatment method applied, the concentration of soluble COD increased after the pretreatment process. According to Figure 1a, the COD solubilization increased with temperature and decreased with heating rate. For CH pretreatment, the maximum (DS = 26.3%) solubilization was measured at a temperature of 160 °C and a thermal heating rate of 3 °C/min, while minimum (DS = 5.4%) solubilization was measured at a temperature of 80 °C for a heating rate of 11 °C/min. The results of the COD solubilization using the MW and RF pretreatments under various temperatures and holding times are compared in Figure 1b. Increasing the pretreatment temperature and holding time had statistically significant effects on COD solubilization. As per Figure 1b, the maximum (DS = 17.1%) and minimum (DS = 7.0%) COD solubilization were obtained under the pretreatment temperatures of 120 °C and 60 °C and holding times of zero and 120 min, respectively. Consistent with the results of the COD solubilization tests, the solubilization of sugar, protein, and humic acid were increased by increasing the pretreatment temperature and holding time and decreasing the heating rate.

**Figure 2:**
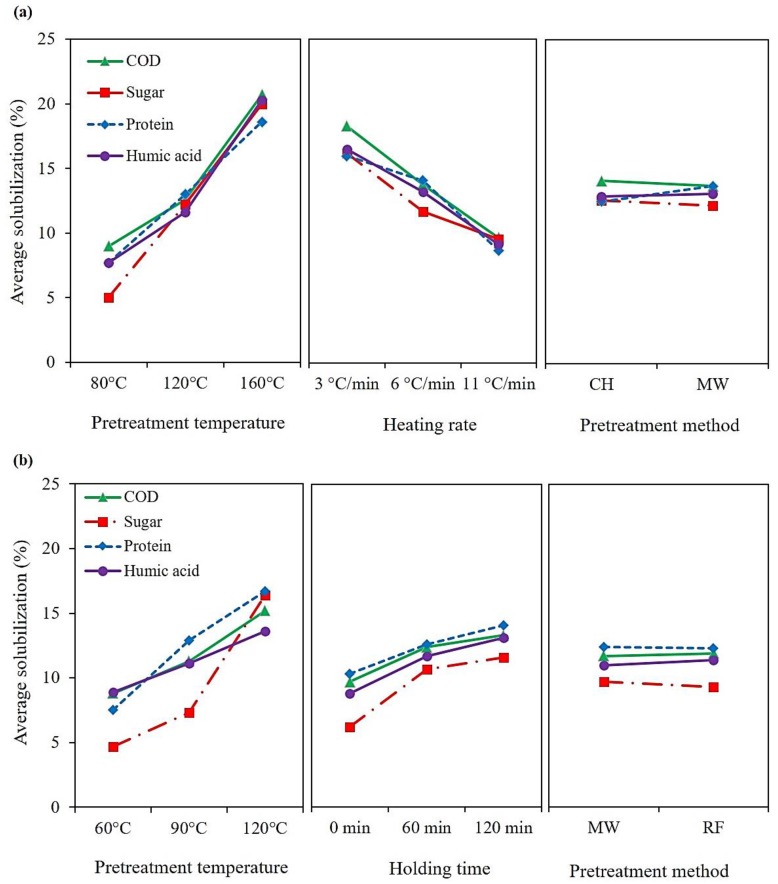
The main effect plot of COD, sugar, protein, and humic acid solubilization from; (**a**) CH vs. MW study; (**b**) MW vs. RF study.

In Table 1, the *p*-values associated with each of the experimental independent variables are shown. There was no statistically significant difference among the three pretreatment methods (CH, MW, and RF) in terms of COD and biopolymers solubilization (*p*-value > 0.05). The main effect plots of the COD, sugar, protein, and humic acid solubilization associated with the “CH vs. MW” and “MW vs. RF” studies are shown in Figure 2a,b respectively. As observed from Figure 2, despite the significant effects of pretreatment temperature, heating rate, and holding time on COD and biopolymers solubilization, there was no significant difference among the application of different thermal pretreatment methods (CH, MW, and RF) for sludge solubilization.

It should be mentioned that the findings of other research studies evaluating the effects of pretreatment temperature, heating rate, and holding time are in agreement with those of this study [10,24,25,26,27]. However, in terms of the effect of pretreatment method (CH vs. MW), it is difficult to derive conclusions about any possible differences between CH and MW pretreatment methods due to the significant inconsistency among the results of the published research [9,11,13,14,15,21,28]. Because under identical thermal profile, the three different pretreatment methods compared in this research achieved the same level of sludge solubilization, it is inferred that the main reason behind the contradictory results of the previous research is the inability to maintain identical thermal profiles among thermal pretreatment methods. Considering the statistically significant effects of the final temperature, heating rate, and holding time on sludge disintegration, any comparison among thermal pretreatment methods should be conducted under identical thermal profiles. Otherwise, it may result in unreliable and contradictory conclusions as observed in the literature [9,11,13,14,15,17,18,19,20,21].

### 2.2. Comparison of CH, MW, and RF Pretreatments for Bioenergy Production

Following the solubilization tests, a series of mesophilic and thermophilic batch digesters were set up to compare the bioenergy production from the municipal sludge that was pretreated with CH, MW, and RF methods. According to the results obtained through the “CH vs. MW” study, except a few pretreatment scenarios which were conducted under the highest heating rate of 11 °C/min, thermal pretreatment increased the bioenergy production compared to the non-pretreated sludge samples. Consistent with the results of the solubilization tests, statistically significant effects of the pretreatment temperature and heating rate were also observed on the production of bioenergy through the mesophilic and thermophilic AD of municipal sludge (*p*-value < 0.05). It was also proven that both the CH and MW pretreatment methods can achieve similar bioenergy production if they are applied under identical thermal profiles (*p*-value > 0.05).

**Figure 3:**
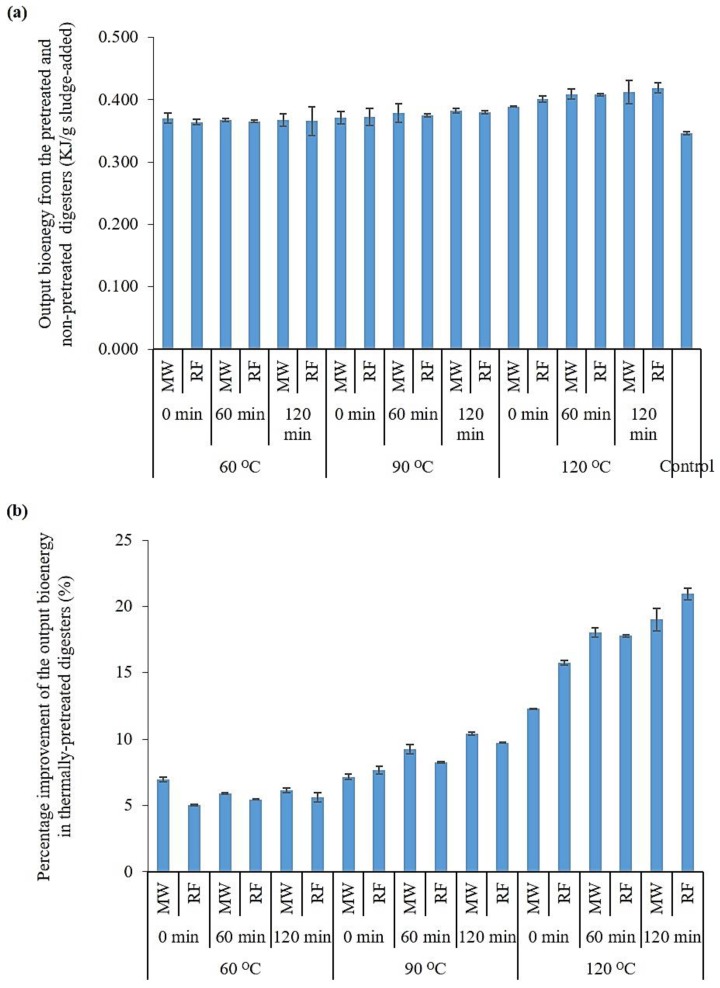
(**a**) Output energy from the pretreated and non-pretreated digesters; (**b**) percentage improvement (relative to the control digester) in output energy.

Figure 3a compares the bioenergy production of the digesters fed with MW-, RF-, and non-pretreated sludge in a unit of kJ/g sludge-added. The percentage improvements in the bioenergy production from the thermally-pretreated digesters (compared to the control digester) are also shown in Figure 3b. According to Figure 3b, all digesters fed with thermally-pretreated sludge produced a higher amount of bioenergy (in the form of methane) compared to the control (non-pretreated) digesters. The maximum bioenergy production (0.419 kJ/g sludge-added) was obtained using the RF pretreatment at a temperature and holding time of 120 °C and 120 min, respectively. Depending on the condition of the pretreatment applied (temperature and holding time), the output energy of the pretreated digesters was increased 5 to 21% compared to the control digester.

The statistical analysis revealed that both the pretreatment temperature and holding time had statistically significant effects on the bioenergy production (*p*-value < 0.05). However, no statistically significant difference was found among the digesters fed with MW- and RF-pretreated sludge (*p*-value > 0.05) in terms of the output energy. These results further confirm the outcomes of the solubilization study in which the three thermal pretreatment methods were proven to have similar effects on the improvement of sludge solubilization. The overall statistical analysis revealed that if the thermal profile is identical, the type of the pretreatment method used (CH, MW, and RF) is not a significant factor determining the production of bioenergy through the digestion of municipal sludge. This outcome is in contrast to that of the previous studies in which one of the thermal pretreatment methods (i.e., CH and MW) is suggested as a superior method over another for improved bioenergy production [13,14,15,21]. 

**Figure 4:**
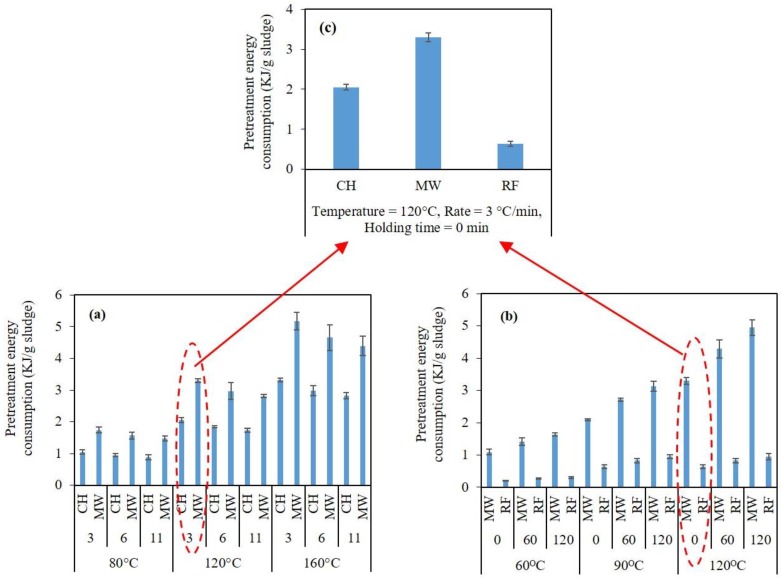
Electrical energy consumption (input energy) during different pretreatment condition; (**a**) CH vs. MW; (**b**) MW vs. RF; (**c**) CH vs. MW vs. RF.

As per Figure 3, the overall trend of the thermal pretreatment effect on the bioenergy production was that the higher the pretreatment temperature or heating rate is, the higher the output energy of the digesters is. However, due to higher electrical energy consumption, the net energy ($E_{net}$) of the sequential pretreatment/AD system may not necessarily be higher at elevated pretreatment temperatures. Figure 4 compares the electrical energy consumption of the CH, MW, and RF pretreatment methods. As per Figure 4a, under any pretreatment condition (thermal profile) used, the MW pretreatment system consumed 56–66% more electrical energy compared to the CH system. According to Figure 1b, regardless of the thermal pretreatment condition used, the energy consumption during the MW pretreatment was significantly (229–441%) higher than that of the RF pretreatment.

In this research, the “CH vs. MW” comparison was performed under different pretreatment temperatures and heating rates and a fixed holding time (0 min). On the other hand, the “MW vs. RF” comparison was conducted under a fixed heating rate (3 °C/min) and various pretreatment temperatures and holding times. Therefore, Figure 4c compares the electrical energy consumption of the pretreatment systems during a given pretreatment condition at which all the three systems were used. Under this pretreatment condition (temperature = 120 °C, heating rate = 3 °C/min, holding time = 0 min), the CH, MW, and RF pretreatment systems consumed 2.0, 3.3, and 0.6 kJ electrical energy per gram of sludge, respectively (Figure 4c).

It has been already demonstrated that under an identical thermal profile, the bioenergy production of the digesters fed with thermally-pretreated sludge is independent of the type of the pretreatment system used. Therefore, the lower the input energy consumption of the pretreatment system is, the higher the net energy production of the sequential pretreatment/AD process will be.

**Figure 5:**
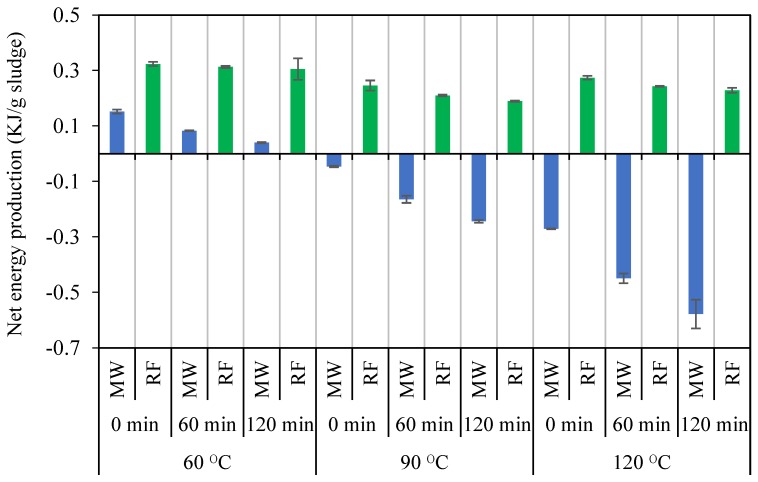
The net energy production of the MW- and RF-pretreated digesters.

The net energy of an advanced AD system calculated from Equation (2) does not include the amount of the thermal energy that can be recovered from the pretreated sludge before feeding to the digester. The recovered thermal energy can be used to preheat the sludge, increase its temperature to some extent, and therefore reduce the input energy of the system. An efficiency factor of 75–90% for the thermal energy recovery via a heat exchanger is suggested by other researchers in the field [29,30,31,32]. In this study, an efficiency factor of 80% was selected. Figure 5 compares the net energy production through the advanced AD process utilizing the MW and RF pretreatment system. As shown in Figure 5, due to high electrical energy consumption (input energy), the MW pretreatment resulted in a negative energy balance for the pretreatment temperatures of above 60 °C, but, the application of the RF pretreatment achieved a positive net energy balance under all the pretreatment conditions tested. 

Despite the positive net energy production achieved via sequential pretreatment/AD process utilizing RF heating, the net energy increase via methane generation still stayed below the energy input requirement for RF heating. Therefore, the control (non-pretreated) digester had the highest net energy production. Given the secondary benefits of thermal hydrolysis of municipal sludge established in the literature such as improved pathogen destruction and faster dewaterability [33,34], the results of the current research conducted under batch flow regime warrant a more comprehensive energy analysis with data generated from larger scale continuously fed digesters (simulating full-scale AD more closely) using RF pretreatment on thickened sludge. The application of RF heating on thickened sludge at much higher solids concentrations (i.e., > 10% total solids (TS), as seen in patented thermal hydrolysis processes) will expect to achieve higher net energy than the control digesters. This outcome will be significant considering the fact that according to Cano et. al. (2015), despite the enhanced solubilization or biogas production achieved, almost all of the pretreatment technologies consuming electricity cannot satisfy their energy requirement [35]. Energy analyses from continuous-flow AD studies incorporating RF pretreatment of thickened sludge are currently underway at UBC Bioreactor Technology Group. 

## 3. Materials and Methods

### 3.1. Municipal Sludge Characteristics

Table 2 summarizes the main characteristics of the thickened waste-activated sludge (TWAS) and dewatered sludge cake (DWSC) which were used in this research. The sludge samples were collected from the City of Kelowna’s municipal wastewater treatment plant (WWTP) located in the Okanagan Valley in the southern interior of the Province of British Columbia, Canada. At Kelowna’s WWTP, the wastewater undergoes physical treatment processes (i.e., screening, grit removal, and primary sedimentation) followed by a biological nutrient removal (BNR) system. The WAS produced through the BNR process is collected from the bottom of secondary clarifiers and sent to a dissolved air flotation unit for thickening. The generated TWAS is mixed with the fermented primary sludge (PS) at a ratio of 67%-TWAS to 33%-PS by volume. The mixed sludge is transferred to a centrifuge unit and dewatered to produce the DWSC.

### 3.2. Thermal Pretreatment Systems

#### 3.2.1. CH Pretreatment System

**Figure 6:**
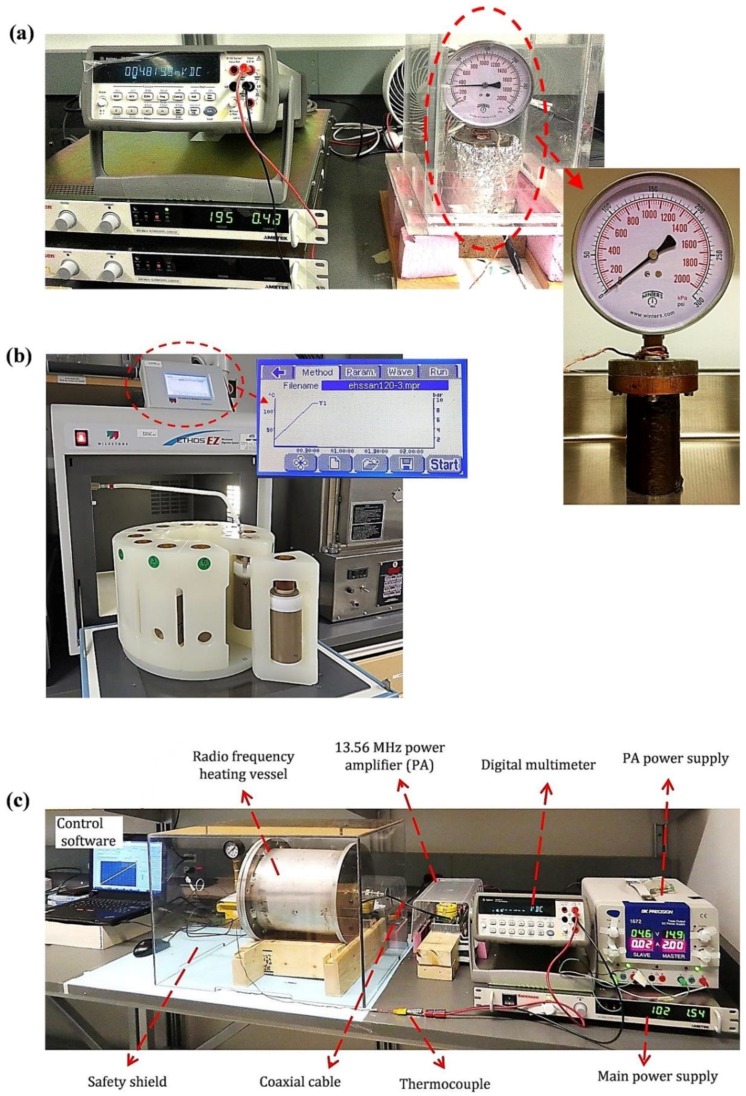
Thermal hydrolysis systems; (**a**) conventional (conductive) heating system (**b**) 2.45 GHz microwave oven; (**c**) 13.56 MHz radio frequency heating system.

Figure 6 shows the configuration and the major components of the three electricity-based thermal pretreatment systems compared in this research. As shown in Figure 1a, the CH system consists a custom-built pressure-sealed vessel. The other components of the CH system include a thermocouple (type K), safety valve, pressure gauge (Winters PEM Series), external fiberglass insulator, DC power supply (Sorensen, Ametek, San Diego, CA, USA), digital multimeter (Agilent, 34401A, Santa Clara, CA, USA), control software, and safety shield. The pressure-sealed vessel was made of a copper cylinder with height, diameter, and thickness of 9.2, 3.8, and 0.32 cm, respectively. The copper vessel was wrapped with 1.5 m of a 0.3 mm-diameter nichrome wire (#80/20) and had a total electrical resistance of 500 Ω. The voltage of the DC power supply was controlled with a computer equipped with a custom-developed LabVIEW program. The heating profile was controlled by changing the DC voltage applied to the nichrome heater. 

#### 3.2.2. MW Pretreatment System

As shown in Figure 6b, a bench-scale 1.2 kW oven operated at a commonly used frequency of 2.45 GHz (ETHOS-EZ, Milestone Inc., Sorisole, Italy) was used for MW pretreatment. The MW system was capable of heating 1.2 L of sludge to a maximum temperature and pressure of 300 °C and 35 bar, respectively. The heating profile in the MW oven was controlled by measuring the temperature of the load using an ATC-400-CE thermocouple.

#### 3.2.3. RF Pretreatment System

The RF heating system is shown in Figure 6c and was custom-designed based on the electrical properties of municipal sludge [36]. The RF heating vessel consisted of a parallel plate structure enclosed in a dielectric cylinder. The cylinder was machined from a solid piece of Teflon which has very low dielectric loss and the parallel plate structure created a uniform electric field throughout the load volume. The Teflon vessel was surrounded by an aluminum cylinder to provide RF shielding from the electric field. A 1 kW RF generator operating at a frequency of 13.56 MHz was connected to the RF heating applicator. The system could heat 400 mL of sludge up to a temperature of 160 °C and under heating rates up to 15 °C/min. A closed loop control system was used to control the thermal profile in the load. A thermocouple was immersed in the load cylinder and the RF power applied to the load was controlled by changing the DC supply voltage to the generator. A software program running in LabVIEW periodically sampled the load temperature and adjusted the DC voltage to maintain a specific software defined thermal profile. The software provided a convenient way to control thermal ramp rates and final load temperatures to match heating profiles used in CH and MW experiments. Further details on the electrical design of the RF heating system are available in other publications [37,38,39].

### 3.3. Experimental Design

#### 3.3.1. CH vs. MW Comparison

The comparison of the thermal hydrolysis systems was made through a series of solubilization tests followed by AD assessment. Table 3 shows the independent variables and their levels included in the design of the experiments. For the CH vs. MW comparison study (Table 3a), the experimental design included a wide range of final temperatures (80, 120 and 160 °C) and heating rates (3, 6 and 11 °C/min). As listed in Table 3a, fourteen different combinations of the independent variables (pretreatment method, heating rate, and final temperature) were evaluated in addition to one control scenario (without pretreatment). After the solubilization study, a fully randomized half-factorial design was used to define the experimental combinations for the mesophilic and thermophilic batch AD. As a result, 27 mesophilic batch digesters (including triplicates) with pretreated sludge and inoculum were set up. The same number of digesters (27) were also set up under the thermophilic condition. Also, one set of blank digesters (only set up with inoculum) and one set of control digesters (with non-pretreated sludge and inoculum) were included in the experiment. A total of 66 batch digesters (including triplicates) were operated simultaneously.

#### 3.3.2. MW vs. RF Comparison

The comparison of CH and MW pretreatment methods showed that there was no statistically significant difference between the two methods in terms of sludge solubilization and digester performance under identical thermal profiles. Based on this outcome, the next set of experiments compared MW and RF heating methods. MW heating was conducted using the same apparatus and the same experimental methodology in both sets of experiments (CH vs. MW and MW vs. RF), and the MW heating results obtained in both sets of experiments were consistent. Therefore, although the experiments described in the paper were carried out in two phases, the methodology was identical and the outcomes of the experiments were compared. Further, both sets of experiments included control digesters to provide benchmarks for comparison with and without thermal pretreatment.

For the RF vs. MW comparison study (Table 3b), the experimental design included one control and 18 combinations of three independent variables including pretreatment method (RF vs. MW), final temperature (60, 90 and 120 °C), and holding time (0, 60, and 120 min). Although a temperature of 60 °C was not expected to have a significant effect on sludge solubilization and subsequent AD processes, it was included in the experimental design to investigate any possible non-thermal (athermal) effects of the MW and RF pretreatments. Experimental results to compare CH with MW heating, which are summarized in Table 3a, show that a low thermal ramp rate of 3 °C/min resulted in the best sludge disintegration and biogas production. Based on the outcome of the first experiments comparing CH and MW heating, subsequent experiments to compare heating methods, including MW and RF heating, used a heating rate of 3 °C/min. Following the solubilization study, 63 mesophilic batch digesters were set up to compare the effect of the two pretreatment methods (MW vs. RF) on AD performance.

### 3.4. Sludge Disintegration Study

The effects of CH, MW, and RF pretreatments on sludge disintegration were evaluated by comparing the soluble concentration of COD and biopolymers (i.e., sugar, protein, and humic acid) before and after thermal pretreatment. The Standard Methods procedure (Section 5250 D) on the application of closed reflux colorimetric method was followed in measuring the COD concentration [40]. The procedure proposed by Dubois et al. (1956) was used for sugar analysis [41]. The COD and sugar measurement was done using an Evolution 60S UV-Vis spectrophotometer (Thermo Fisher Scientific, Inc., Waltham, MA, USA) at the wavelength of 600 and 490 nm, respectively. Protein and humic acid quantification was done at a wavelength of 750 nm with a multi-detection microplate spectrophotometer (BioTek Synergy HT, Winooski, VT, USA). The protein and humic acid sample preparation was done following the modified Lowry’s method [42]. 

### 3.5. Anaerobic Digestion Study

The batch AD experiments were initiated by placing the substrate and inoculum into 160 mL-bottles. The mesophilic inoculum was taken from a pilot-scale digester which has been continuously fed with a mixture of primary and secondary sludge from the Kelowna’s WWTP at a sludge retention time (SRT) of 20 d for more than three years. The thermophilic inoculum was taken from a full-scale digester located at the Annacis Island WWTP in Vancouver (BC, Canada) utilizing a mixture of primary and secondary sludge. The substrate/inoculum mixing ratio was calculated based on the food to microorganism ratio (F/M) of 2.1 ± 0.2 (g VS/g VS). To keep the digester pH above 6.5 throughout the digestion process, additional alkalinity (4000 mg/L of ${\mathrm{CaCO}}_{3}$) was added into each digester in the form of potassium bicarbonate and sodium bicarbonate. The inoculum was degassed for a period of one week prior to the start of the batch assays. Upon mixing the inoculum and substrate, and before sealing, the digesters were purged with nitrogen gas. The mesophilic and thermophilic batch digesters were placed in two separate incubators (Innova, 44R, Eppendorf Canada, Mississauga, ON, Canada) set at 90 rpm and a temperature of 35 °C and 55 °C, respectively. The value of cumulative bioenergy production of the batch digesters with a duration time of 35 d was used to determine the bioenergy production from the batch digesters.

### 3.6. Energy Analysis

As shown in Equation (2), the net energy of an advanced AD system (pretreatment +AD) can be determined by subtracting the amount of energy consumed during the sludge pretreatment (input energy) from the amount of energy generated as methane (output energy).

(2) $$\;E_{net}=E_{out}-E_{in}$$

where, $E_{net},\;E_{out}\;\mathrm{and}\;E_{in}$ are the system net energy, output energy and input energy, respectively. In this research, the $E_{out}$ of the digesters was determined considering the methane energy content of $55.6\;\frac{\mathrm{kJ}}{{g\;\mathrm{CH}}_{4}}$ and the density of$\;0.715\;\frac{g}{L}$ at standard temperature and pressure (0 °C, 1 atm) [5]. To determine the$\;E_{in}$, the voltage and the current supplied to the pretreatment systems (CH, MW, and RF) were continuously recorded during the entire pretreatment period. The input power ($P_{t}$) was then determined by multiplying the recorded current and voltage. The total energy consumption ($E_{in}$) of the systems was then calculated by integrating the power over the entire heating time as follows:

(3) $$\;E_{in}=\int_{0}^{T}P_{t}dt\;$$

For the CH and RF pretreatment systems, the current and voltage of the DC power supply were automatically recorded by a computer equipped with a custom-developed LabVIEW program. However, for the MW system, it was more convenient to measure the input voltage and current to the MW oven using an oscilloscope which was connected to the AC line input to the oven. It is noteworthy that a complete energy analysis of advanced AD system would need to consider other processes in the AD such as mixing or the energy to thermally regulate the temperature of the sludge inside the digester. However, these additional energy factors are assumed to the same for non-pretreated (control) and thermally-pretreated AD systems and have therefore been excluded from the energy equation.

### 3.7. Analytical Method

The total and volatile solids (TS and VS) concentration were determined following the procedures of the Standard Methods (Sections 2540 B and 2540 E) [40]. The ammonia (NH_3_-N) analysis was conducted using an electrode connected to a dual channel pH/ion meter (Accumet Excel XL25). The total volatile fatty acids (VFAs) were measured in the form of acetic, propionic, and butyric acids by injecting the samples into an Agilent 7890A gas chromatograph (GC) using an autosampler. The GC utilized an Agilent 19091F-112 capillary column with a length 25 m and a diameter of 320 µm. It was also equipped with a flame ionization detector (carrier gas flow rate: 25 mL·He/min; oven, inlet, and outlet temperatures: 200, 220 and 300 °C, respectively). Before injecting samples into the GC, the samples were centrifuged for 20 min at 10,000 rpm and then filtered through 0.45 µm membrane filters. The volume of the biogas was measured using a manometer. The biogas composition was determined in the form of ${\mathrm{CH}}_{4},{\;\mathrm{CO}}_{2},{\;\mathrm{and}\;N}_{2}$ gases using an Agilent 7820A GC equipped with an Agilent G3591-8003/80002 packed column and a thermal conductivity detector (oven, inlet, and outlet temperatures: 70, 100 and 150 °C, respectively).

### 3.8. Statistical Analysis

The statistically significant effects of the input parameters (i.e., pretreatment method, temperature, heating rate, etc.) were evaluated by multi-factor ANOVA at a 95% confidence level (α = 0.05) using Minitab Software 17 (Minitab Inc., State College, PA, USA). The Fisher’s least significant difference test was applied to compare all pairs of means. The Anderson-Darling test was used to judge if the data follow normality distributions. The sample preparation was done randomly following a randomized experimental order determined by Design-Expert 9 software.

## 4. Conclusions

According to the results and analyses, under identical thermal profiles, the method of thermal pretreatment (CH, MW, and RF) was not a significant factor determining the sludge disintegration and AD performance. The input energy measurements revealed that the CH and MW pretreatment methods consumed 100–440% more electrical energy than the RF heating system to achieve the same pretreatment conditions. The RF heating system used in this study was designed to heat municipal sludge efficiently and therefore it demonstrates the importance of the heating applicator design. Based on the results of the energy analysis, the energy consumption during pretreatment using all thermal hydrolysis methods (CH, MW, and RF) was higher than the increase in the net bioenergy which was achieved during the AD process in form of methane. This resulted in higher net energy production in the control (non-pretreated) digester compared to the sequential pretreatment/AD process. As a way of reducing the input energy per unit dry mass of the sludge, thermal hydrolysis can be applied on thickened sludge at higher solids concentrations (i.e., > 10% TS). In addition, a more representative energy analysis can be carried out on the data collected from larger scale continuously fed digesters which simulate full-scale AD. Given these results, the authors are currently conducting a more comprehensive energy analysis from continuous-flow AD studies incorporating RF pretreatment.

## Figures and Tables

**Table 1:** Summary of the *p*-values obtained via an overall statistical analysis.

Variable	Levels	COD	Sugar	Protein	Humic Acid
Temperature (°C)	80, 90, 120, 160	0.000	0.000	0.000	0.000
Rate (°C/min)	3, 6, 11	0.000	0.000	0.000	0.000
Holding time (min)	0, 60, 120	0.019	0.000	0.002	0.013
Method	CH, MW, RF	0.321	0.317	0.512	0.770

**Table 2:** The characteristics of the municipal sludge used in this research *.

Description	Thickened Waste-Activated Sludge	Dewatered Sludge Cake
pH	6.5 ± 0.1	5.8 ± 0.2
TS (% *w*/*w*)	3.5 ± 0.2	19.2 ± 0.34
VS (% *w*/*w*)	2.7 ± 0.2	16.8 ± 0.35
VS/TS (%)	77.4	87.6 ± 0.24
Total COD (mg/L)	37,420 ± 574	265,702 ± 9422
Soluble COD (mg/L)	1740 ± 350	11,991 ± 591
Total VFAs	309 ± 23	1857 ± 36
Ammonia (mg/L)	201± 17	678 ± 82
Alkalinity (mg/L as CaCO_3_)	632 ± 128	2145 ± 327

* TS: Total solids; VS: Volatile solids; COD: Chemical oxygen demand; VFAs: Volatile fatty acids as summation of acetic, propionic, and butyric acids.

**Table 3:** The experimental design used for comparison of CH, MW, and RF pretreatment systems.

(a) CH vs. MW Experimental Design				(b) MW vs. RF Experimental Design			
Method	Temperature (ᵒC)	Rate (ᵒC/min)	Digester type	Method	Temperature (ᵒC)	Holding time (min)	Digester type
CH	80	11	Batch mesophilic	MW	60	0	Batch mesophilic
	120	6				60	
		11				120	
	160	3			90	0	
MW	80	3				60	
		6				120	
	120	3			120	0	
	160	6				60	
		11				120	
CH	80	6	Batch thermophilic	RF	60	0	
		11				60	
	120	3				120	
	160	3			90	0	
		6				60	
MW	80	3				120	
	120	6			120	0	
		11				60	
	160	11				120	
